# Identification of Protein Networks Involved in the Disease Course of Experimental Autoimmune Encephalomyelitis, an Animal Model of Multiple Sclerosis

**DOI:** 10.1371/journal.pone.0035544

**Published:** 2012-04-17

**Authors:** Annelies Vanheel, Ruth Daniels, Stéphane Plaisance, Kurt Baeten, Jerome J. A. Hendriks, Pierre Leprince, Debora Dumont, Johan Robben, Bert Brône, Piet Stinissen, Jean-Paul Noben, Niels Hellings

**Affiliations:** 1 Biomedical Research Institute, Hasselt University and Transnationale Universiteit Limburg, School of Life Sciences, Hasselt, Belgium; 2 VIB – Bioinformatics Training and Service Facility (BITS), Gent, Belgium; 3 GIGA-Neuroscience, University of Liège, Liège, Belgium; 4 Biochemistry, Molecular and Structural Biology, Katholieke Universiteit Leuven, Heverlee, Belgium; University of Lyon, France

## Abstract

A more detailed insight into disease mechanisms of multiple sclerosis (MS) is crucial for the development of new and more effective therapies. MS is a chronic inflammatory autoimmune disease of the central nervous system. The aim of this study is to identify novel disease associated proteins involved in the development of inflammatory brain lesions, to help unravel underlying disease processes. Brainstem proteins were obtained from rats with MBP induced acute experimental autoimmune encephalomyelitis (EAE), a well characterized disease model of MS. Samples were collected at different time points: just before onset of symptoms, at the top of the disease and following recovery. To analyze changes in the brainstem proteome during the disease course, a quantitative proteomics study was performed using two-dimensional difference in-gel electrophoresis (2D-DIGE) followed by mass spectrometry. We identified 75 unique proteins in 92 spots with a significant abundance difference between the experimental groups. To find disease-related networks, these regulated proteins were mapped to existing biological networks by Ingenuity Pathway Analysis (IPA). The analysis revealed that 70% of these proteins have been described to take part in neurological disease. Furthermore, some focus networks were created by IPA. These networks suggest an integrated regulation of the identified proteins with the addition of some putative regulators. Post-synaptic density protein 95 (DLG4), a key player in neuronal signalling and calcium-activated potassium channel alpha 1 (KCNMA1), involved in neurotransmitter release, are 2 putative regulators connecting 64% of the identified proteins. Functional blocking of the KCNMA1 in macrophages was able to alter myelin phagocytosis, a disease mechanism highly involved in EAE and MS pathology. Quantitative analysis of differentially expressed brainstem proteins in an animal model of MS is a first step to identify disease-associated proteins and networks that warrant further research to study their actual contribution to disease pathology.

## Introduction

MS is an inflammatory autoimmune disease of the central nervous system (CNS) in which genetic, environmental and immunological factors are involved [1], [2]. The disease is characterized by blood brain barrier (BBB) breakdown, demyelination, oligodendrocyte apoptosis, progressive axonal damage and reactive astrogliosis [3]–[5]. These pathological hallmarks are present in the multifocal inflammatory lesions of the CNS, primarily localized in the white matter. The infiltration of autoreactive T cells, B cells and macrophages, and the production of pro-inflammatory cytokines are known to take part in the formation of inflammatory CNS lesions [4], [6]–[9]. Still, the exact cause and underlying molecular mechanisms remain poorly understood, but are crucial in the search for new therapeutic options. A proteomics approach was chosen to get more insight in the molecular processes of MS.

Proteomics studies are valuable to get an overview of protein expression in cells, tissues or organisms. These protein expression profiles can provide indications towards molecular mechanisms involved in normal and disease processes. In the past, gel-based proteome studies of brain [10] and cerebrospinal fluid (CSF) [11]–[14] were carried out by comparison of intensities of (silver) stained gel spots, a procedure that may suffer from experimental variability and poor reproducibility. Only adequate quantitative approaches will allow the analysis of disease processes over time in the brain or CSF during neuroinflammation. Two-dimensional fluorescence difference gel electrophoresis (2D-DIGE) is a very sensitive gel-based proteomics technique that is unique through the utilization of fluorescently labelled samples on the same gel, and the application of an internal standard for intra- and inter-gel comparisons and normalization.

In MS research, some quantitative proteomics studies have already been completed [15]–[17]. Looking at 2D-DIGE experiments, mostly biomarker studies on human CSF were performed [18]–[22]. In one proteomics study in MS research, a comparison between multiple plaque-types was performed to obtain new therapeutic targets [23], although not by 2D-DIGE. Post-mortem MS samples are often a snapshot of longstanding disease. Therefore, a well characterized homogeneous animal model, experimental autoimmune encephalomyelitis (EAE), was selected for this study to obtain a sample of the inflammatory lesions. Only 2 experiments were published in which CNS tissue of EAE animals was used for a 2D-DIGE study [24], [25]. In these studies protein expression was compared between two experimental groups. To get a better understanding of the pathomechanisms in MS and EAE, we decided to use experimental groups at different time points during the disease.

Here we report disease stage-specific variations in brain protein expression found in samples from different time points during acute EAE, a well characterized animal model of MS. The brainstem of this model was selected to focus on CNS inflammatory pathways involved in the lesion development and regulation of EAE, as it was shown that disease related macrophage infiltration at the onset of acute Lewis rat EAE was mainly localized to the caudal part of the brainstem [26]. We performed a 2D-DIGE study to quantitatively compare protein levels at different disease stages. Samples were obtained before onset of the symptoms, at the top of the disease and after recovery. This allows us to create graphs of brain protein levels over time. We were able to identify 75 unique proteins present in 92 differential gel spots. All of these proteins were analyzed with Ingenuity Pathway Analysis (IPA) software to disclose connections between these proteins, and thus define pathways that could be involved in the molecular mechanisms of MS.

## Results

### EAE brain proteome analysis by 2D-DIGE/mass spectrometry

**Figure 1:**
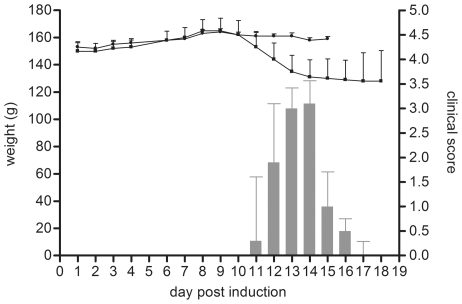
Clinical scores and weight changes of EAE and control animals. EAE was induced by injection of MBP in CFA (bars and squares). Control animals were CFA injected (dots). The controls showed no clinical symptoms. Each value represents the mean ± standard deviation of n animals: control day1-15, n = 3; EAE day1–9, n = 9; day10–14, n = 6 and day15–18, n = 3.

**Figure 2:**
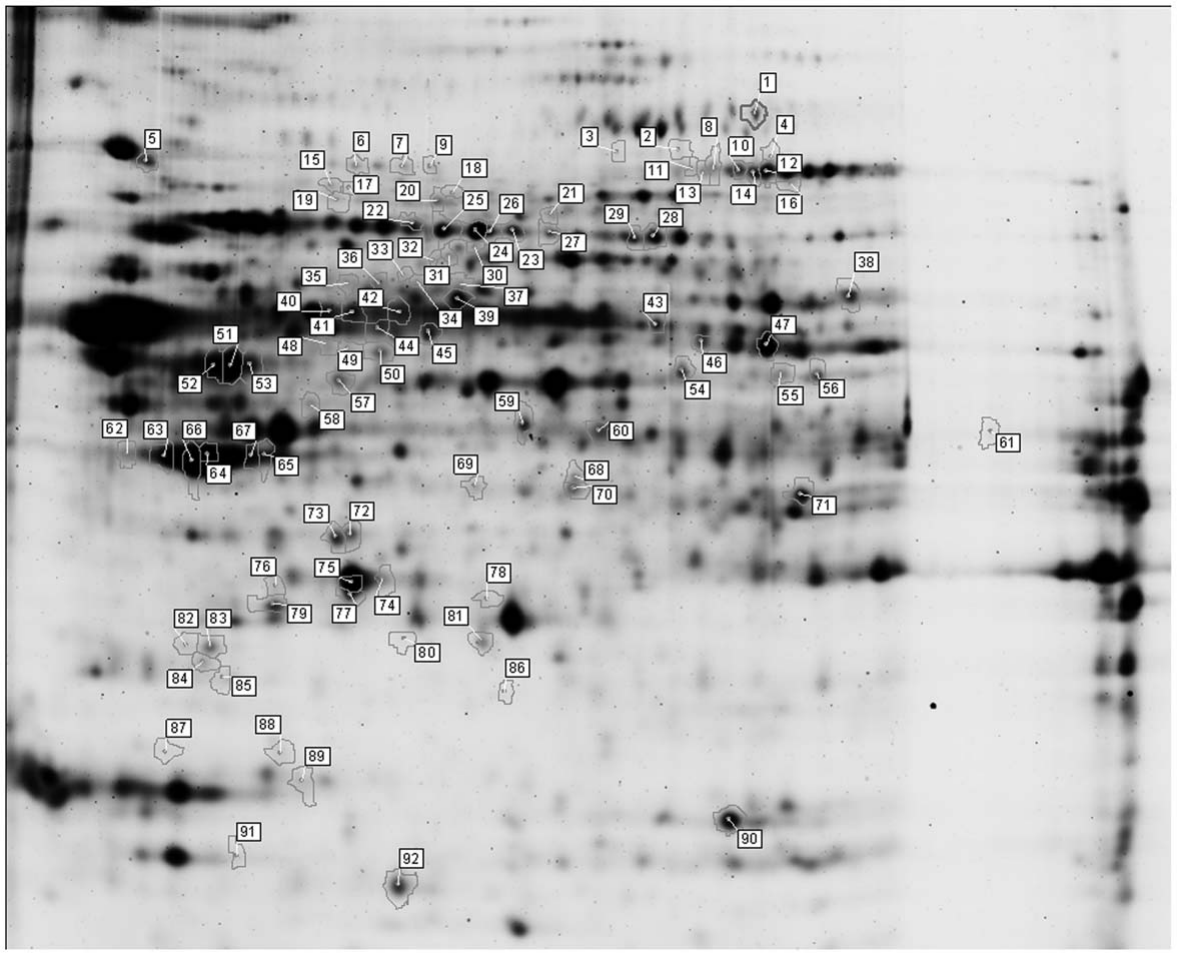
2D-DIGE gel image. The 92 spots presented have a shift in abundance over the four experimental conditions (control, disease onset, top, and recovery) (ANOVA ≤ 0.05). Spots were picked from preparative 2D-gels and proteins identified by nano-LC-ESI-mass spectrometry. The proteins were identified with significant MASCOT and SEQUEST scores. Spots are numbered as in Table S1.

Detergent-soluble protein extracts were isolated for a quantitative 2D-DIGE study to identify differential proteins in the brainstem of EAE-animals and controls at different stages of the disease. Controls were CFA injected, whereas acute EAE was induced by immunization with myelin basic protein (MBP) (Figure 1). The EAE animals were divided into three groups: before onset, at the top, and following recovery of the disease. We identified proteins in 92 differential gelspots (ANOVA≤0.05) with DeCyder 7.0 gel analysis software and nano-LC-mass spectrometry (Figure 2). The difference in fluorescence intensity, as reported in the DeCyder software, indicates a change in expression, turnover and/or protein modification. Sixty-nine of the 92 differential protein spots were even more stringently regulated (ANOVA ≤ 0.01, bold in Table S1). A total of 130 proteins were identified in these 92 spots, since multiple proteins can be present in one gelspot (Table 1). Furthermore, 24 of these proteins were present in multiple (2–10) spots. Overall, 75 unique proteins were identified.

**Table 1:** Identification of differentially expressed protein spots (A≤0.05).

# protein identifications/spot	# identified spots	# proteins
1	63	63
2	22	44
3	5	15
4	2	8
Total	92 identified spots	130 identified proteins

**Figure 3:**
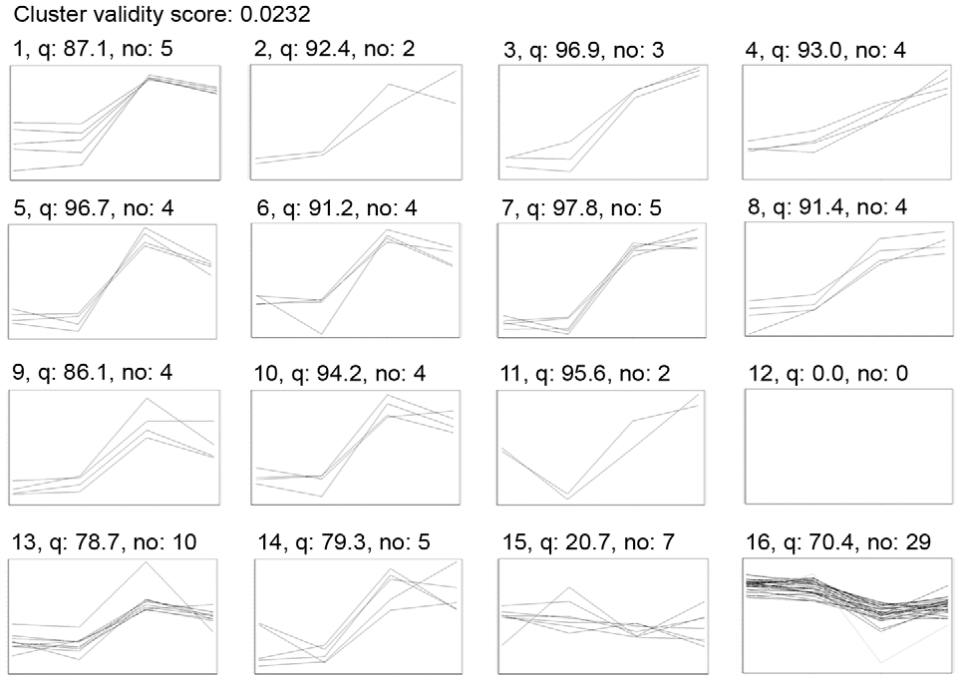
SOM analysis. A SOM analysis was performed to group spots with a similar expression pattern, in this way clustering the spots that are regulated in the same way. Sixteen clusters were obtained, one (12) containing no spots. The X-axis chronologically displays the experimental groups (C-O-T-R) while the Y-axis displays the log standardized abundance (scale is not identical for the different clusters). The cluster number, quality value (q) and number of spots present in the cluster (no) are indicated above the graphs.

To get a better view on the expression profile of these spots along the disease course, a cluster analysis was performed. Using self organizing maps (SOM) analysis, spots that have the same expression patterns during the disease are grouped together (Figure 3). The average ratio and T-test, for the six possible comparisons between the four conditions included in this study, provided detailed information on the time course of the proteome changes induced in the inflamed brain (Table S2).

### Identity and validity of differential proteins

**Figure 4:**
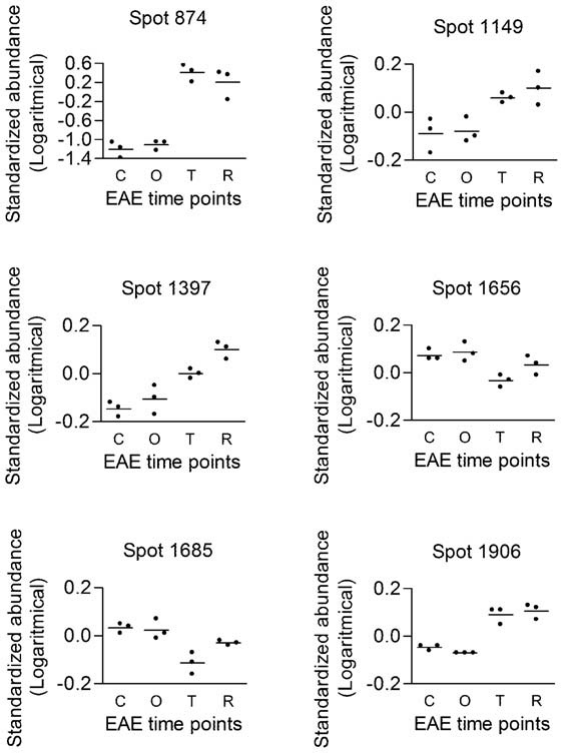
Protein expression patterns over the disease course. 2D-DIGE expression profiles: ALB (spot 874), PDIA3 (spot 1149), GFAP (spot 1397), CNP (spot 1656 and 1685) and CAPG (spot 1906). The log standard abundance (the relative abundance change normalized to signals in internal standard specific for each spot) is indicated for control, onset, top and recovery samples.

**Figure 5:**
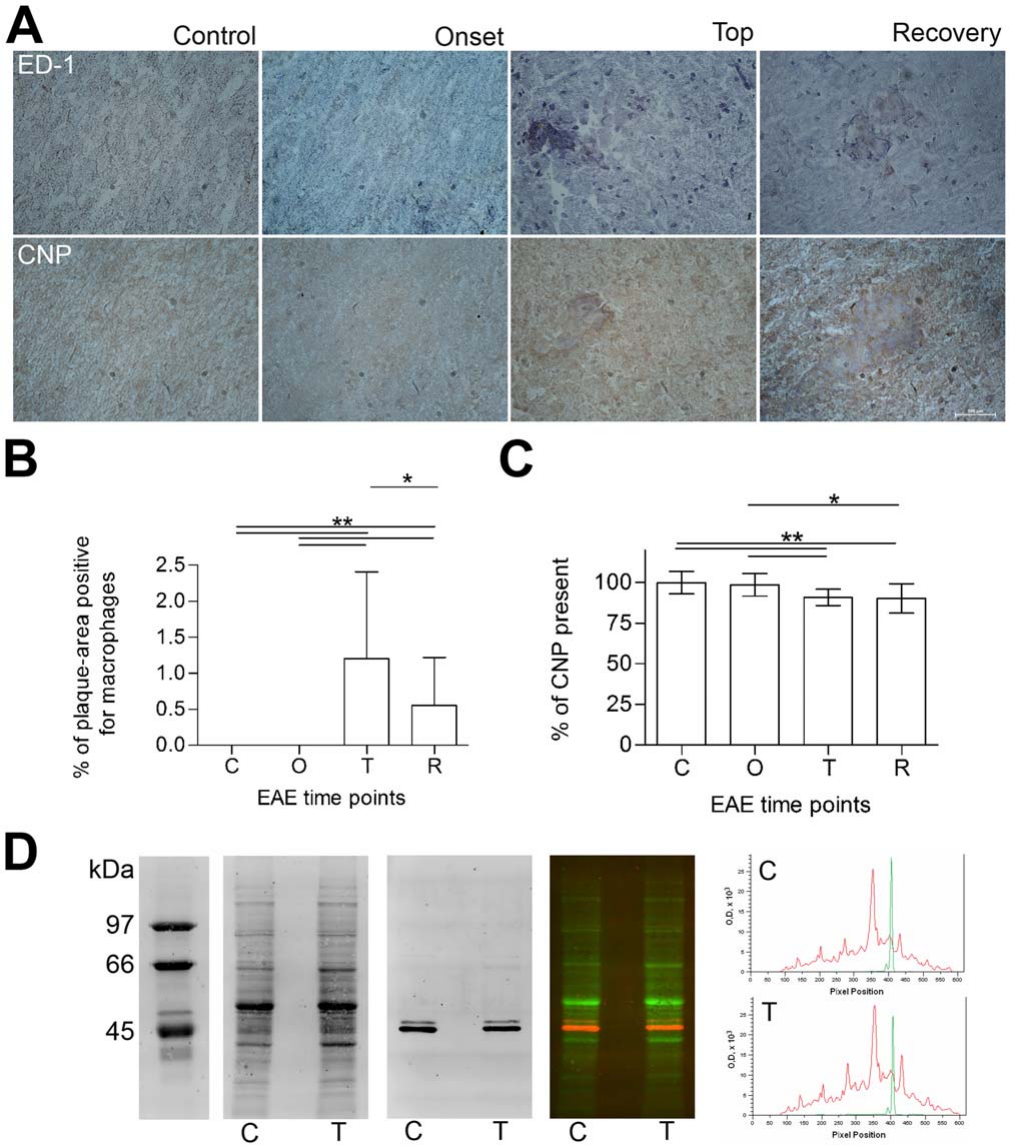
Validation of the 2D-DIGE results. Immunohistochemistry was performed to demonstrate the presence of macrophages and CNP. Macrophage (ED-1) and CNP immunostaining of rat spinal cords (same animals as for 2D-DIGE) from control, and EAE rats before disease onset, top and recovery are shown in panel A. These IHC stainings were quantified (Panel B and C), and expression levels compared by Dunn's multiple comparison test (GraphPad Prism4). The error bars indicate standard deviations of measurements performed at least in triplicate. *: significant difference, p<0.01 and **: significant difference, p<0.001. In Panel D, a quantitative 1D CNP immunoblot of EAE brainstem homogenate from control and disease top is shown. An overview of the fluorescent total protein staining, anti-CNP immunostaining, the fluorescent overlay of both (red and green overlay), and finally a representation of the fluorescent signals as processed with ImageQuant TL software (GE Healthcare). The red curve corresponds with the total protein content and the green curve with the CNP fluorescence. Both a representative control animal (c) and one at the disease top (t) are presented.

BBB disruption, astrocyte activation and macrophage infiltration are processes known to occur in MS and acute EAE. We focused on proteins related to these disease processes to verify the experimental setup and analyses. Indeed, serum albumin (ALB, e.g. spot 874), glial fibrillary acidic protein (GFAP, e.g. spot 1397) and macrophage-capping protein (CAPG, spot 1906) represent these pathological hallmarks and are all upregulated at the top of the disease (Figure 4). An ED-1 macrophage staining on spinal cord slices verified the infiltration of macrophages in the CNS (Figure 5A). In contrast to the absence of macrophages in controls and just prior to disease onset, macrophage infiltration was significantly increased at the top of the disease (Figure 5B), a similar pattern as seen for CAPG. Identification of differential proteins that represent processes actively involved in the disease, verifies our experimental design and the ability to pick up disease-related proteins.

The expression pattern of 2′,3′-cyclic-nucleotide 3′-phosphodiesterase (CNP), an abundant myelin protein, was significantly decreased in the inflamed brain (Figure 4, spot 1656 and 1685). This could be indicative for myelin loss. We confirmed this CNP decrease with immunohistochemistry (IHC) and western blot (WB) (Figure 5). IHC was performed and quantified at the site of inflammation. CNP was significantly decreased at the top of the disease and after recovery (Figure 5C). Furthermore, a fluorescent quantitative anti-CNP WB revealed two bands, consistent with CNP1 (46 kDa) and CNP2 (48 kDa). Both CNP1 and CNP2 were significantly decreased at top of the disease compared to control animals (fold change −1.54±0.03 and −1.40±0.05 respectively). In conclusion, with two independent techniques, we were able to confirm the 2D-DIGE expression data for CNP.

### Principal component analysis and Ingenuity Pathway Analysis

**Figure 6:**
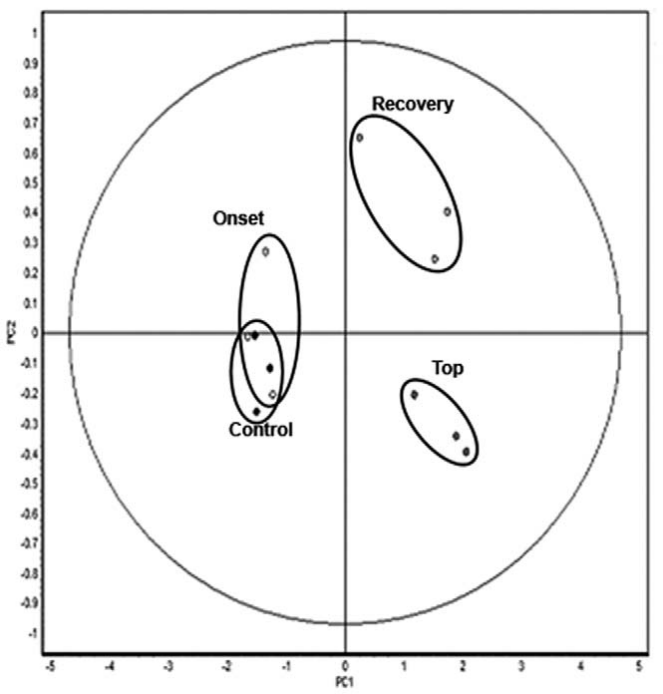
Unsupervised multivariate analysis discriminating between early and late groups. PCA reduces the dimensionality of a multidimensional analysis and displays the two principle components that can distinguish between the two largest sources of variation within the dataset (92 spots, ANOVA ≤ 0.05). Principle component analysis clustering the 12 individual spotmaps into the four conditions by two principle components: PC1, which distinguishes 90% of the variance, and PC2 distinguishes an additional 3.8% of the variance.

Principal component analysis (PCA) is an unsupervised multivariate method used to analyze the variability between experimental groups. A dimension reduction is applied previous to classification, reducing the possibly correlated variables (differential spots) to a set of uncorrelated variables. In this way a principal component represents a linear combination of the differential spots. Each sample (spotmap) is represented in the PCA plot with respect to the principal components. A PCA was performed on our dataset and shows clustering of the samples according to the disease stage. A clear separation between the early disease stage (before onset of the disease) and the late disease stages (top and recovery) is evident (Figure 6). Furthermore, samples from the top of the disease are separated from recovery samples. In contrast, the control and onset samples were not separated; implicating that differences in brain protein expression were not sufficient for separation between these two conditions.

**Figure 7:**
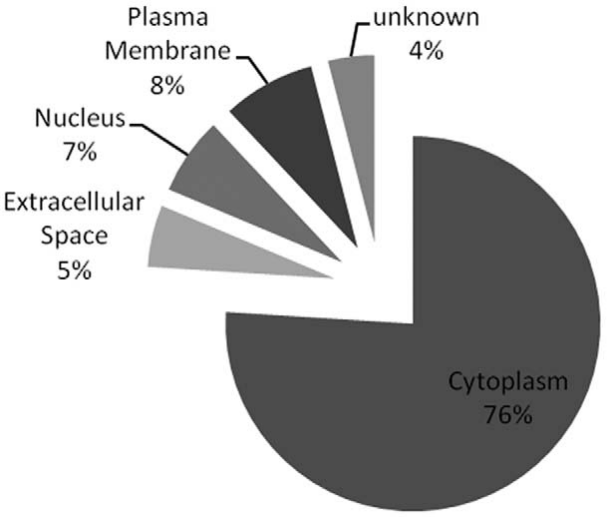
GO-Compartments. The 75 unique proteins (ANOVA ≤ 0.05) were categorized according to the subcellular compartment (extracellular space, plasma membrane, cytoplasm, nucleus, and unknown). Information was collected from Gene ontology by IPA. Percentages are presented.

Human homologues of the 75 unique proteins identified here were subsequently analyzed with IPA, a software tool capable of mapping proteins onto existing networks and pathways. Cellular compartments as designated by IPA (gene ontology based) indicated that the majority of identified proteins (76%) were cytoplasmic in origin (Figure 7). Mapping of our proteins onto biological pathways and disease networks demonstrated that 16 proteins were linked to nervous system development and function (p-value: 2.47E-05–4.50E-02), and that 53 of the 75 proteins were associated with neurological disease (p-value: 1.35E-15–4.50E-02). Post synaptic density protein 95 (DLG4) and amyloid precursor protein (APP) appear to be central points in the IPA networks identified here, but they were not detected in the 2D-DIGE study.

**Figure 8:**
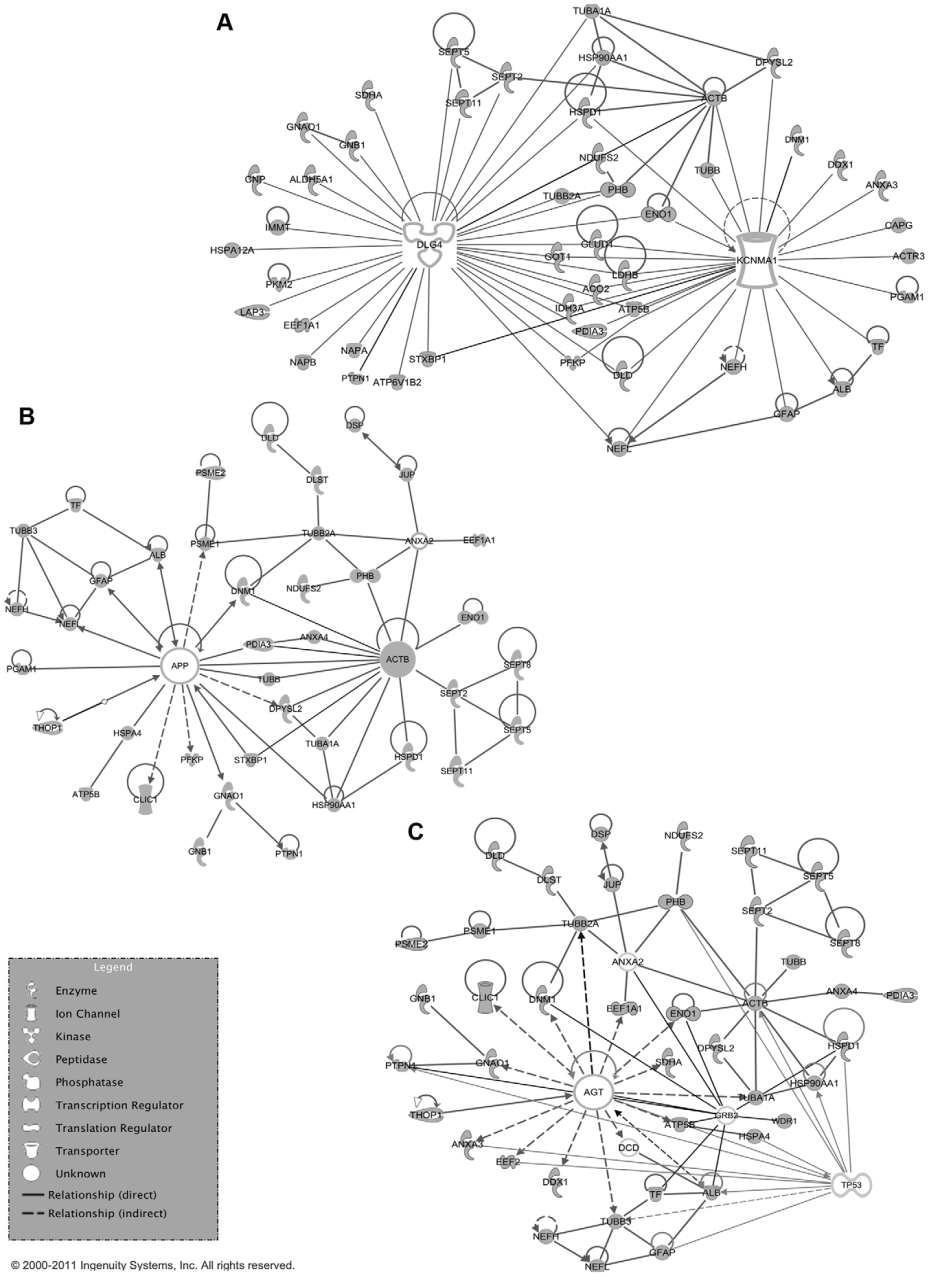
Ingenuity pathway analysis networks build with focus proteins. The DLG4-KCNMA1 network (Panel A), APP-ACTB network (Panel B) and AGT-TP53 network (Panel C) are represented. These networks were obtained using the IPA-KB by linking proteins from the data-set (75 unique proteins) to the focus proteins. Nodes containing proteins identified in the dataset have a grey fill.

Another possibility in the IPA software was the comparison of the 75 unique proteins to a list of MS-related proteins present in the IPA knowledge base. As expected due to technical restrictions, only one of these mostly membrane-associated MS-related proteins was also identified in our 2D-DIGE study, protein disulfide-isomerase A3 (PDIA3, spot 1149) (Figure 4). However, fifty-eight of our differential proteins were linked to these MS-related proteins, mostly by downstream biochemical pathways (data not shown). Angiotensin (AGT) and calcium-activated potassium channel alpha 1 (KCNMA1) were MS-related proteins from the IPA knowledgebase with a strong relation to our data. Together with DLG4 and APP, they were selected for building focus networks of our dataset. These networks suggest an integrated regulation of the identified proteins with the addition of some putative regulators. They are a model for the effects of these four proteins on our dataset (Figure 8 and Table S3). Forty-eight proteins (64%) of the 2D-DIGE dataset were directly linked to DLG4 and/or KCNMA1. Forty-two (56%) were linked to AGT and 40 (53.3%) to APP. In the AGT network another important potential regulator of our dataset was identified, being tumor protein p53 (TP53).

**Figure 9:**
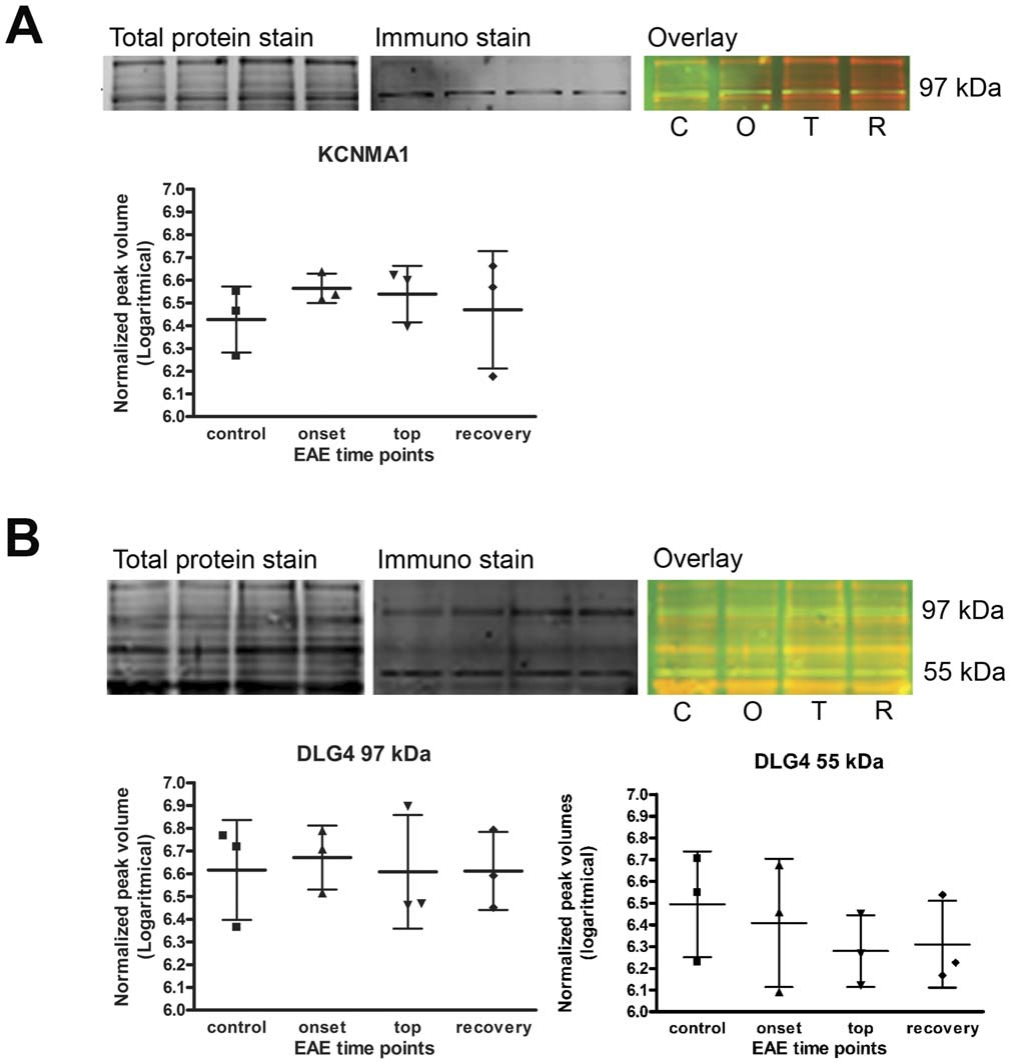
Western blot analysis of DLG4 and KCNMA1. A quantitative fluorescent western blot was performed to analyze the presence and expression levels of KCNMA1 (Panel A) and DLG4 (Panel B). By means of peak detection, the normalized peak volumes were used for quantification. No significant difference was found in expression levels, but both proteins were detected in the samples of the 2D-DIGE experiment. All animals were included in the WB analysis; control (C), onset (O), top (T) and recovery (R).

**Figure 10:**
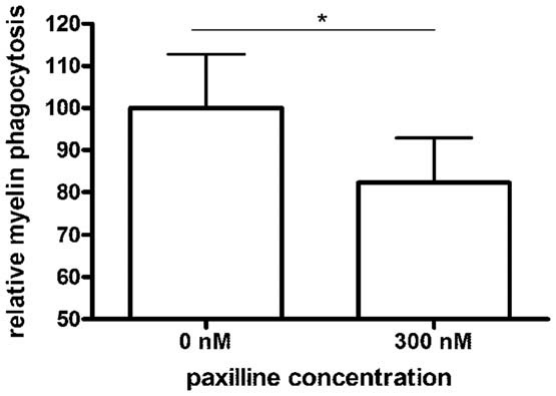
Myelin phagocytosis assay. The influence of paxillin, a specific blocker of the KCNMA1 channel, on myelin phagocytosis was studied to evaluate the possible biological involvement of this protein in EAE and/or MS related disease processes. After macrophage activation, myelin and paxillin were added and phagocytosis was measured by flow cytometry.

We confirmed the presence of DLG4 and KCNMA1 in our brain samples performing a quantitative fluorescent immunoblotting (Figure 9). By using a combination of a total protein staining and an immunostaining on western blot, it is possible to correct for differences in total protein loading. Fluorescent stains allow for peak detection and quantification. For both DLG4 and KCNMA1, presence of the protein in our samples could be established, but no expression differences were detected between disease stages. This in part explains why these proteins were not picked up in our 2D-DIGE analyses. Even though they are not differentially expressed, their presence in the centre of the IPA networks suggests a role for DLG4 and KCNMA1 as central regulators in the molecular mechanisms of disease progression. KCNMA1 is a calcium-activated potassium channel with a direct connection to CAPG, a protein involved in actin-based cell motility and thus important for macrophage functions such as migration and myelin phagocytosis, processes known to be highly involved in MS and EAE pathology [7]. We demonstrated that specific blocking of KCNMA1 using paxillin significantly reduced myelin phagocytosis by LPS activated macrophages (−17.79±10.67%, p<0.01, Figure 10). This nicely illustrates that the central regulators reported here indeed have a functional role in the disease process.

## Discussion

We identified proteins present in 92 differential spots of the inflamed brain of EAE animals by means of a comparative 2D-DIGE proteomics analysis. Changes in the abundance of these 92 spots can discriminate between early (before onset) and late (top and recovery) disease stages by PCA of their values in the sample spotmaps. Seventy-five unique proteins were identified in these 92 differential spots by means of mass spectrometry. Some of these proteins represent known disease processes such as BBB disruption (ALB), astrocyte activation (GFAP) and macrophage infiltration (CAPG). Others are not yet linked to MS, and warrant further research. An in-depth network evaluation was performed for all 75 unique proteins.

Seventy percent of our identifications (53/75 proteins) are part of the biological pathway of neurological disease. One such example is the decrease in GABA transaminase (ABAT, spot 1437 and 1439), succinate-semialdehyde dehydrogenase (ALDH5A1, spot 1428) and mitochondrial glutamate dehydrogenase 1 (GLUD1, spot 1316 and 1331). Both ABAT and ALDH5A1 are enzymes responsible for the degradation of GABA, the principal inhibitory neurotransmitter of the CNS that is also involved in inflammation [27]. When these enzymes decrease, GABA levels will increase. GLUD1 is an enzyme responsible for the interconversion of glutamate and alpha-ketoglutarate. A decrease in GLUD1 may result in an increase of glutamate, a neurotransmitter reported to be involved in MS excitotoxicity [9] and the precursor of GABA. Overall, these proteome changes indicate an increase of GABA at the top of the disease. Dysregulation of the GABA pathway in MS brains has been described in several studies and the implications of these findings have been tested in EAE [27], [28].

Four central network-nodes were suggested by IPA. DLG4 and APP are nodes from the IPA networks of our data, while KCNMA1 and AGT are MS-related proteins in the IPA knowledge base, with a strong relationship to our data. APP is an integral membrane protein that is concentrated at neuronal synapses. It can be synthesized by microglia, not only in response to direct nerve injury but also in immune-mediated disease such as EAE [29]. A role for APP in immune and repair mechanisms of the CNS is suggested [29]. A second network is build around AGT, a protein produced by astrocytes in the brain [30]. It is part of the renin-angiotensin system (RAS) that affects the immune response in general and the neuroinflammatory processes in the context of EAE [31]. TP53, a well-known tumor suppressor that responds to cellular stress and can induce apoptosis and changes in metabolism is also present as a regulator in this network. TP53 was recently described to be an important ‘network-hub’ that interacts with a lot of genes associated with MS, indicating a role for this protein in the disease, namely the expansion of autoimmune cell clones [32]. Sixty-four percent of our data are connected to DLG4 and/or KCNMA1, which highlights their possible role in EAE/MS. DLG4 is a membrane-associated protein implicated in the clustering of receptors, ion channels and associated signaling molecules in the post-synaptic membrane. It is a key player in neuronal signaling. KCNMA1 is an IPA MS-related protein involved in neurotransmitter release. The channel activity increases during hypoxia and decreases in response to reactive oxygen species (ROS) [33]. Mitochondrial ion channels play an important role in cellular events such as apoptosis (caused by increased mitochondrial membrane permeability), exocytosis and synaptic transmission and are believed to contribute to cytoprotection [33]. Both DLG4 and KCNMA1 are key regulation proteins of mitochondrial enzyme complexes involved in the cellular response to oxidative stress, a process that is also described in EAE/MS [34]. We were able to detect DLG4 and KCNMA1 in our samples, but they were not differential between the experimental groups. This could indicate that the activation of these molecules does not influence their expression. The alterations in their downstream molecules however suggest that these pathways are activated during the different disease stages and thus involved in disease mechanisms. Indeed, DLG4 immunoreactivity in both gray and white matter of EAE spinal cord tissue was reciprocally associated with damage of postsynaptic structures and directly associated with disease activity. When EAE animals were in remission, DLG4 expression partly restored, further emphasizing the actual involvement of the DLG4 pathway in disease [35]. Furthermore, we showed that specific blocking of KCNMA1 in macrophages decreased the ability of macrophages to phagocytose myelin, a pathological hallmark of MS and EAE lesions. The above documented functional role of DLG4 and KCNMA1 in EAE indicates that these networks could be biologically important for MS pathology and warrants further research.

Previously a quantitative iTRAQ study was reported comparing EAE spinal cord proteome between EAE and control animals [17]. Six proteins were identified in common with our study (ALB, ANXA3, LAP3, PDIA3, PMSE2 and TF). These 6 proteins show an increased abundance during the disease in the iTRAQ study as well as in our study. Han et al. [23] performed a proteomic study on MS tissue, comparing different MS lesion types. They reported a total of 2302 proteins related to MS plaques of which 158, 416 and 236 proteins were unique to acute plaques, chronic active plaques and chronic plaques respectively. Sixty-four of the 75 unique proteins in our study were also reported in the study of Han et al., all but one (DSP is only present in chronic active lesions) are common for the different lesion types. The 11 unique proteins of our study are ACTB, ACTR3, ANXA3, EEF1A1, GNB1, Ifi47, LOC674678, PTPN1, STXBP1, TUBA1A and TUBB1. We believe that all 75 proteins reported here warrant further evaluation, as the data of Han et al. in contrast to our data are not quantitative and non of our 75 proteins were functionally evaluated.

The analysis of the brainstem proteome during EAE identified significant differences in the levels of proteins involved in mitochondrial energy production, apoptosis, antioxidant activity, cytoskeleton regulation and the immune system. The in depth network analysis by IPA as described here adds a major value to 2D-DIGE studies and the combination of both technologies is a prerequisite to find common regulators that extend even the limitations of the proteomics technology. Some proteins may play central roles through functional regulation without being differentially expressed. Still, IPA analysis helps to reveal pathways involved in the disease process and thereby also potentially involved proteins that were not picked up directly by 2D-DIGE analysis. This strategy helps generate new hypotheses and to select unknown targets in pathways with relevance to MS.

In conclusion, the brain proteome study as presented here identified biological events involved in neuroinflammation that may be important during EAE, and also in MS. IPA analysis provides network information on the differentially expressed proteins in the 2D-DIGE study and enables the detection of proteins that cannot be picked up by a gel-based technology due to the technical restrictions favoring soluble, mostly cytoplasmic proteins [36], [37]. The focus on disease-related networks in this work enables us to select several relevant topics in MS for further validation studies in animal models and MS patients and possibly allows the selection of targets for therapy.

## Materials and Methods

### Sample Collection

EAE was induced in 7 week-old female Lewis-rats by subcutaneous immunization with myelin basic protein (MBP) in Complete Freunds Adjuvant (CFA) [26]. Animals were weighted and scored daily according to the following scale 0, no neurological abnormalities; 0.5, partial loss of tail tonus; 1, complete loss of tail tonus; 2, hind limb paresis; 3, hind limb paralysis; 4, moribund; 5, death. Before disease onset (9 dpi), at the top (14 dpi) and after recovery (18 dpi) three animals were sacrificed and transcardially perfused to obtain blood-free brain stems (5 mM EDTA in PBS pH 7.2 with Complete Protease Inhibitor (Roche)). Control animals (n = 3) were injected with CFA only and sacrificed at 15 dpi. After isolation, all tissues were frozen in liquid nitrogen and subsequently stored at −80°C. This study was in strict accordance with the EU legislation, Directive 86/609/EEC. The protocol was approved by, and carried out in strict agreement with the recommendations of, the local Ethical Committee for Animal Experiments of Hasselt University (permit number: 201023).

### Protein Extraction

Proteins were extracted as described by Sizova et al. [38]. Briefly, brainstems were lyophilized, crushed (Kontes tissue grinder) and proteins extracted before ultracentrifugation. Samples were then desalted and the buffer was exchanged to labeling buffer (7 M urea, 2 M thiourea, 4% w/v CHAPS in 30 mM Tris HCl pH 8.5) using ultrafree®-MC PLCC centrifugal filter units (Millipore, cut-off 5 kDa). Protein concentration was determined using the 2D Quant kit (Amersham Biosciences) and aliquots were stored at −80°C.

### Labeling

Minimal labeling with N-hydroxysuccinimidyl-ester dyes Cy2, Cy3 and Cy5 (GE healthcare) was performed as described by the manufacturer (Ettan™ DIGE Basic course, GE healthcare) with some minor adaptations. Labeling of 50 µg of proteins was accomplished with 300 pmol of Cy3 or Cy5 in dimethylformamide (DMF, Acros organics). The pooled internal standard, containing identical protein amounts from all samples, was labeled with Cy2. To avoid dye specific labeling artifacts, there was a dye swap in each group (three samples from any condition were never labeled all with Cy3 or Cy5).

### 2D-GE

For isoelectric focusing (IEF), a 3–10 NL IPG strip of 24 cm (GE Healthcare) was rehydrated for 8 hours. IEF runs (IPGphor 3, GE Healthcare) and preparation of second dimension SDS-PAGE gels was done according to the manufacturer's Ettan™ DIGE Basic course (GE healthcare). Strip equilibration was carried out with equilibration buffer I and II (6 M urea, 2% SDS, 50 mM Tris pH8.8, 0.02% bromophenol blue and 30% glycerol) containing 1% DTT or 4.5% iodoacetamide respectively. After equilibration, strips were mounted onto the SDS-PAGE gels (12.5%), and run for 2 hours at 5 mA/gel and overnight at 25 mA/gel in the Ettan DALTsix electrophoresis system (GE healthcare). 2× SDS electrophoresis buffer was used in the lower buffer chamber and 3× SDS electrophoresis buffer in the upper buffer chamber.

### 2D-DIGE analysis

CyDye-labeled 2D-DIGE gels were scanned on the Ettan DIGE imager (GE healthcare). Gel images from all three CyDyes were loaded into DeCyder 7.0 software (GE healthcare) and analyzed. Statistical significance was calculated using Student's t test and analysis of variance (ANOVA) to compare the variation in abundance within a group to the magnitude of change between groups. Spots present in 85% of the gel images, and with a statistically significant ANOVA (p≤0.05) were considered for further analysis. Unsupervised principal component analysis (PCA) was performed using the DeCyder extended data analysis (EDA) module.

### Spotpicking and protein digestion

For spot picking (Ettan SpotPicker, GE healthcare) a preparative gel was made containing 200 µg of an unlabeled sample and 50 µg of the labeled internal standard. Bind-silane (GE healthcare) and reference stickers were applied on the glass plate containing spacers before pouring the gel, thereby ensuring the accuracy of robotic protein excision. In-gel digestion using trypsin (Promega) was performed manually as described by Shevchenko [39].

### Mass spectrometric analysis and protein identification

The mass spectrometer was calibrated and tuned as described in LCQ ‘Operator's Manual’ Revision B July 1996. Instrumental ion optics were further optimized for analysis of doubly charged peptide ions by direct infusion (1 µl/minute) of synthetic peptide ‘IFGKGTTLSVSSNIQ’ at 10 pmol/µl in 0.1 M acetic acid ([M+2H]^2+^ = 776.42). Tryptic digests were dried *in vacuo*, solubilized in 20 µl 0.1 M acetic acid in water containing cortisol (4 pg/µl) as an internal standard and analyzed in data-dependent mode by nanoflow HPLC/ESI(+)-MS/MS [40]. Stability of the chromatographic process and ESI efficiency were monitored using cortisol base peak m/z 361.2. Bovine serum albumin (10 fmole BSA on-column) was used for analytical system control. LCQ Xcalibur v2.0 SR2 raw files and spectra were selected from within Proteome Discoverer 1.0.0.43 (Thermo Electron) with following settings: minimal peak count, 50; total intensity threshold, 4000; and S/N, 6. Peak lists were searched with Sequest v1.0.43 and Mascot v2.2.0.2 against the EMBL-EBI International Protein Index database for rat (v3.69; 39578 entries) and for mouse (v3.69; 56737 entries) both with following settings: fragment tolerance, 1.00 Da (monoisotopic); parent tolerance, 3.0 Da (monoisotopic); fixed modifications, carbamidomethylation of cystein; variable modifications, oxidation of methionin; max missed cleavages, 2. Outcome of both search engines was validated with Scaffold v.3.00.03 (Proteome Software) with minimum peptide and protein probability set to 95% and 99.9% respectively. The protein identifications thus returned by Scaffold for each gel spot were manually validated considering spectral quality.

HASH in TRANCHE representing our data: nq+91eLffYILUDgesGy6Hx/mmDF6a7hPyvCMAdFcKwBUafN2Dr6DUlM0HaKPWb5XYiVh/nbmTKuRAL+sxbLFD4FyzTwAAAAAAAAEcQ =  = 

### Immunohistochemistry

Tissue sections (10 µm, Leica CM1900 UV microtome) of the spinal cord were used for IHC. Macrophages were detected with the ED-1 staining (mouse anti-rat CD68, AbD serotec) as a primary antibody (1/200, 2 hours). Myelin protein 2′, 3′-cyclic nucleotide 3′-phosphodiesterase (CNP) was detected using mouse anti-CNP (Millipore) as a primary antibody (1/200, overnight). Biotinylated polyclonal rabbit anti-mouse IgG (Dako) was selected as secondary AB (1/400, 1 hour). 10% rabbit serum was chosen for blocking and 3,3′-diaminobenzidine (DAB) for staining. Hematoxylin was used as a counterstaining. For the analysis of the staining (Nikon ECLIPSE 80i microscope, NIS elements software, 20× objective), 3 ED-1 stained tissue sections were scanned for positive staining regions. These regions were defined as inflammatory plaques, and pictures were taken of these areas. Macrophage infiltration was defined as the percentage of positive area in these pictures. For the CNP staining, 3 slices adjacent to the 3 slices stained for macrophages were used. CNP was measured in the area corresponding to the inflammatory plaques in the ED-1 stained slices. Statistical analysis of the difference in macrophage infiltration and CNP expression between the experimental groups was done using Dunn's multiple comparison test (GraphPad Prism4).

### Western blotting

7.5 µg of the brainstem protein extract was separated by 1D SDS-PAGE. After blotting to a nitrocellulose membrane, total protein staining was done by means of ruthenium (II) tris (bathophenantroline disulfonate)(RuBPS) staining (Rubilab) as previously described [41]. A subsequent immunostaining was performed with mouse anti-CNP (Millipore, 1/2500 for 1 hour), rabbit anti-DLG4 (Millipore, 1/1000 for 2 hours) and rabbit anti-KCNMA1 (Millipore, 1/1000 for 2 hours) as primary antibodies. Goat anti-mouse/rabbit Alexa fluor 647 (Invitrogen) were used as a secondary antibody (1/5000, 2 hours). The fluorescent signal was measured with the Ettan DIGE scanner and imagequant TL software was used for processing (GE healthcare). The immunostaining intensities were normalized for unequal protein load (fluorescent total protein stain). The peak volume was used for quantification.

### Network analysis

Mapping of proteins identified by mass spectrometry onto existing networks and pathways was accomplished using Ingenuity Pathway Analysis software (Ingenuity® Systems, www.ingenuity.com). The data set containing protein identities was uploaded into the software. Networks of the identified proteins containing the molecular relationships between genes/gene products were generated algorithmically using the Ingenuity Pathway knowledge base (IPA-KB). Nodes represent genes or gene products and are displayed using various shapes that represent the functional class of the gene product. Nodes are connected by edges (lines) which represent different biological relationships that were in to IPA-KB at the time of creation. In addition, in order to evaluate the overlap of the current work with MS, lists of biological markers were obtained from the IPA-KB as well as from a competitor product GeneGO Metasearch (GeneGo, St. Joseph, MI, USA) and are used for comparison to our data.

Ingenuity Pathway Analysis has been designed to work with human, mouse and rat models. However, in order to support the discussion of MS in human, we decided to use human gene-names in this work. Therefore, all graphs presented in this report contain human nomenclature.

### Myelin phagocytosis assay

Rat macrophages (NR8383 cell line) were cultured in RPMI 1640 medium (Invitrogen) enriched with 10% fetal calf serum (Hyclone, Erenbodegen, Belgium), 50 U/ml penicillin and 50 U/ml streptomycin (Invitrogen). Macrophages were activated with 100 ng/ml lipopolysaccharide (LPS, Sigma) for 18 hours. Next, 100 µg/ml DiI-labeled myelin, isolated and labeled as described previously [42] and paxillin (Sigma) were added for 90 minutes. For paxillin treatment a concentration of 300 nM was used to obtain a total blockage of the KCNMA1 channel [43]. Flow cytometry was used to assess the degree of myelin internalization.

## Supporting Information

Table S1 **Protein identifications.** Differential protein spots were picked and in-gel digestion was performed. Proteins were identified by mass spectrometry.
(XLSX)

Table S2 **Cluster analysis.** A cluster analysis was performed to get a better view on the expression profile of these spots along the disease course. The average ratio and T-test, for the six possible comparisons between the four conditions included in this study, provides detailed information on the time course of the proteome changes induced in the inflamed brain.
(XLSX)

Table S3 **IPA networks.** In this table an overview is presented of all 75 unique proteins and their presence in the IPA networks presented in Figure 8.
(XLSX)
